# Evolution of non-thyroidal illness syndrome in acute decompensation of liver cirrhosis and acute-on-chronic liver failure

**DOI:** 10.3389/fendo.2023.1104388

**Published:** 2023-01-23

**Authors:** Mona-May Langer, Alina Bauschen, Sabrina Guckenbiehl, Sarah Klauss, Teresa Lutz, Gerald Denk, Denise Zwanziger, Lars C. Moeller, Christian M. Lange

**Affiliations:** ^1^ Department for Gastroenterology and Hepatology, University Hospital Essen, University Duisburg-Essen, Essen, Germany; ^2^ Department of Internal Medicine II, Ludwig-Maximilians University (LMU) University Hospital Munich, Munich, Germany; ^3^ Department of Endocrinology, Diabetes and Metabolism, University Hospital Essen, University Duisburg-Essen, Essen, Germany

**Keywords:** liver cirrhosis, systemic inflammation, acute-on-chronic liver failure, thyroxin, non-thyroidal illness syndrome

## Abstract

**Background and aims:**

Non-thyroidal illness syndrome (NTIS) is frequent in critically ill patients and associated with adverse outcomes. We aimed to characterize the evolution of NTIS in patients with acute decompensation (AD) of cirrhosis and acute-on-chronic liver failure (ACLF), since NTIS is not well described in these newly defined syndromes.

**Methods:**

Thyroid hormones (TH) were quantified at baseline in consecutive patients with cirrhosis. In addition, 76 inflammatory mediators were quantified by proximity extension analysis assay in a subgroup of patients. Associations between TH, cirrhosis stage, mortality and inflammation were assessed.

**Results:**

Overall, 437 patients were included, of whom 165 (37.8%), 211 (48.3%), and 61 (14%) had compensated cirrhosis (CC), AD, and ACLF. FT_3_ concentrations were lower in AD versus CC, and further decreased in ACLF. Importantly, NTIS was present in 83 (39.3%) patients with AD and in 44 (72.1%) patients with ACLF (P<0.001). Yet, TSH and TSH-based indexes (TSH/FT_3_-ratio, thyroid index) showed an U-shaped evolution during progression of cirrhosis, suggesting a partially preserved responsiveness of the hypothalamus and pituitary in AD. Infections were associated with lower FT_3_ concentrations in AD, but not in ACLF. Low FT_3_ concentrations correlated significantly with 90-day mortality. Both, AD/ACLF and NTIS, were associated with signatures of inflammatory mediators, which were partially non-overlapping.

**Conclusion:**

NTIS is frequent already in AD and therefore precedes critically illness in a subgroup of patients with decompensated cirrhosis. This might constitute a new paradigm of TH signaling in cirrhosis, offering opportunities to explore preventive effects of TH in AD.

## 1 Introduction

Acute-on-chronic liver failure (ACLF) is defined by acute decompensation of cirrhosis in combination with specific organ failures and consequent high short-term mortality (1). Acute decompensation (AD) in the absence of ACLF-defining organ failures is characterized by better short-term survival rates, although the recent PREDICT study has established subgroups of patients (unstable decompensation, pre-ACLF) with significantly impaired one-year survival (2). New insights into the pathophysiology of AD and ACLF reveal that systemic inflammation is a major determinant of the development of AD (namely unstable AD and pre-ACLF) and in particular of ACLF (2–4). Frequent precipitating events of ACLF such as infections or excessive alcohol consumption are capable to strongly augment cirrhosis-associated systemic inflammation (1). In addition, AD and in particular ACLF are associated with profound metabolic alterations and mitochondrial dysfunction in organs and immune cells, which are most likely causally linked to immune dysfunction and organ failures (5–7).

Triiodothyronine (T_3_) is the bioactive form of thyroid hormones, characterized by a short half-life, while tetraiodothyronine (T_4_) is a prohormone with a half-life of approximately 7 days (8). Secretion of T_4_ from the thyroid is regulated by thyroid-stimulating hormone (TSH) from the pituary gland and thyrotropin-releasing hormone (TRH) from the hypothalamus, the hypothalamus-pituitary-thyroid(HPT)-axis (8). The bioavailability of T_3_ is regulated by three deiodinases, which can convert T_4_ to T_3_ (DIO1 and DIO2) or inactive rT_3_ (DIO3) in tissues (8). Thyroid hormones are essential in maintaining energy metabolism, mitochondrial function, muscle integrity and circulatory function (9). Furthermore, thyroid hormones are involved in the regulation of innate and adaptive immune responses (10). For example, the induction of DIO2 in macrophages, leading to increased TH availability, and increased DIO3 expression in neutrophils, decreasing TH availability, have proinflammatory effects (11). In view of these pleiotropic and central functions of thyroid hormones, it appears plausible that impaired thyroid hormone function contributes to the pathogenesis of AD and ACLF. Indeed, downregulation of T_3_ without upregulation of TSH in critically ill patients is a well-known phenomenon, termed non-thyroidal illness syndrome (NTIS) or low T_3_ syndrome (9). Inflammatory mediators play an important role in the pathogenesis of NTIS, as for example IL-6 or TNF-α can suppress active T_3_ production (12–14). Yet, it remains currently unclear whether reactivation of the thyroid axis improves outcome in critically ill patients (9). NTIS-like conditions have previously been described in patients with liver cirrhosis. For example, low T_3_ levels were associated with poor outcome in intensive care patients with liver cirrhosis (15). Yet, a detailed characterization of NTIS throughout the development of AD and ACLF according to recent definitions is lacking.

In the present study, we therefore aimed to characterize thyroid function parameters and presence of NTIS in patients with the entire spectrum of liver cirrhosis, from compensated cirrhosis to AD and ACLF.

## 2 Patients and methods

### 2.1 Patients

From October 2018 to October 2021, and from December 2021 to May 2022, consecutive patients who were admitted with liver cirrhosis to the University Hospital Essen, Germany, and the Ludwig Maximilian University (LMU) Hospital Munich, respectively, were included in a prospective cohort. While all patients with AD or ACLF were hospitalized as in-patients, most included patients with compensated cirrhosis (146 patients out of 165 patients with compensated cirrhosis) were admitted as outpatients, while some patients with compensated cirrhosis were hospitalized for procedures like liver biopsy or variceal band ligation. Written informed consent was obtained from all participants, and ethical approval was obtained from the local ethical committees (WBE-071). Patients with thyroid disease or thyroid-related medication, pregnant and breast-feeding patients, as well as patients with hepatocellular carcinoma beyond Milan criteria or patients tested positive for Human Immunodeficiency Virus were excluded from the study. AD or ACLF were classified according to the criteria of the Chronic Liver Failure–European Association for the Study of Liver (CLIF-EASL) consortium (1), including sub-stratifications of AD in stable AD, unstable AD and pre-ACLF, as defined in the PREDICT study (2), at admission. Serum samples were collected at baseline, and in most patients during follow-up.

### 2.2 Quantification of thyroid hormone concentrations, definition of NTIS

Serum samples for thyroid function parameters (TSH, free triiodothyronine (FT_3_) and free thyroxine (FT_4_)) were stored at -80°C and accurate temperature was controlled by an in-house master display (Westdeutsche Biobank, University Hospital Essen). Thyroid function parameters were determined on thawed serum samples after recruitment with the Siemens Atellica^®^ IM Analyzer (Siemens Healthineers, Erlangen, Germany). The Atellica^®^ IM TSH3-Ultra Assay is a chemiluminescence immunoassay and according to the product insert the intra-assay variation was <3.6%, the inter-assay variation was <4.5%, the analytical sensitivity was 0.008 mU/L and the detection limit was 0.008 mU/L. The Atellica^®^ IM FT_3_- and FT_4_-Assays are competitive chemiluminescence immunoassays and according to the product inserts the intra-assay variations were <7.6% for FT_3_ and <4.7% for FT_4_, the inter-assay variations were <9.1% for FT_3_ and <6.8% for FT_4_, the analytical sensitivies were 0.31 pmol/L for FT_3_ and 1.3 pmol/L for FT_4_, respectively. The controls of the instrument were performed according to the product inserts (quality control of the manufacturer). Thyroid function parameters are accredited according to DIN EN ISO 15189:2014. NTIS was defined as FT3 concentration below the lower limit of normal (i.e. <3.5 pmol/L) in the absence of disorders of the thyroid gland, according to established definitions (9).

### 2.3 Analysis of inflammatory plasma proteins and mediators by proximity extension analysis assay

Simultaneous quantification of 92 human protein biomarkers in plasma were conducted by Proximity extension analysis assay (Olink, Uppsala, Sweden) in a subgroup of 104 patients with sufficient baseline plasma samples. In total, 76 of the 92 proteins passed quality control and were detected above the limit of detection and were therefore included in the present analysis.

In brief, collected EDTA plasma of patients was thawed and 100 µl shipped on dry-ice to Olink. Protein matched antibodies with unique DNA-tags were bound to specific proteins. Matched DNA-tags hybridized when in near proximity and were extended by PCR. Amplified DNA-tags are detected by qPCR, whereas the qPCR cycles are associated with the relative protein concentration in the sample.

### 2.4 Statistical analysis

Statistical analysis was performed using GraphPad Prism v9.0.2 software (GraphPad Software, San Diego, CA, USA). After testing for Gaussian distribution, two groups were tested by T-test or Wilcoxon-Mann-Whitney-U-Test, as appropriate, while comparison of more groups was done by One-way ANOVA or Kruskal-Wallis-Test, as appropriate. *P* values less than 0.05 were considered to be statistically significant. Associations of outcomes with continuous or dichotomic variables were assessed in linear and logistic regression models, respectively. After univariate analyses, multivariate analyses were performed for significant associations. Multivariate models were obtained by using a *P* value greater than 0.1 for removal from the model. Principal component analyses (PCA) were performed and heat maps build using GraphPad Prism v9.0.2 software.

## 3 Results

### 3.1 Patient characteristics

Overall, 437 patients were included in the present study, of whom 165 (37.8%) patients had compensated cirrhosis, 211 (48.3%) had AD, and 61 (14%) had ACLF. Among patients with AD, 167 (79%), 33 (16%) and 11 (5%) patients had stable AD, unstable AD or pre-ACLF, respectively. Infections at admission were frequent in patients with AD or ACLF (23% and 53%, respectively). During three months of follow-up, 3 (1.8%), 40 (19%), and 19 (31%) of patients with compensated cirrhosis, AD or ACLF died. In addition, 3 (1.8%) patients with compensated cirrhosis, 6 (2.8%) patients with acute decompensation and 5 (8.2%) patients with cirrhosis-ACLF underwent liver transplantation during 3 months of follow-up. Detailed baseline characteristics are shown in **Table 1**.

**Table 1:** Baseline characteristics and laboratory results of included patients.

	Comp. (N=165)	AD (N=211)	ACLF (N=61)	P-value (Comp. vs. AD)	P-value (Comp. vs. ACLF)	P-value (AD vs. ACLF)
General characteristics						
Age [years], mean (SD)	52.1 (12.7)	56.2 (11.7)	55.8 (10.1)	0.03	0.3	>0.99
Male gender; female gender, N (%)	85 (51.5); 80 (48.5)	126 (59.72); 85 (51.52)	39 (63.9); 22 (36.1)	0.1	0.1	0.6
Child Pugh Score, mean (SD)	5.3 (0.5)	8.3 (1.3)	9.5 (1.7)	<0.0001	<0.0001	0.006
CLIF OF score, mean (SD)	6.3 (0.8)	7.5 (1.6)	10.5 (2.1)	<0.0001	<0.0001	<0.0001
MELD score, mean (SD)	10.0 (3.8)	15.7 (5.3)	24.3 (8.2)	<0.0001	<0.0001	<0.0001
Etiology of liver cirrhosis						
Viral, N (%)	27 (16.4)	22 (10.4)	3 (4.9)	0.1	0.02	0.2
NASH, N (%)	9 (5.5)	24 (11.4)	7 (11.5)	0.04	0.1	0.99
Alcoholic, N (%)	49 (29.7)	97 (46.0)	47 (77.1)	0.001	<0.0001	<0.0001
Cholestatic, N (%)	27 (16.4)	24 (11.4)	1 (1.6)	0.2	0.003	0.02
Others, N (%)	53 (32.1)	44 (20.8)	3 (4.9)	0.01	<0.0001	0.004
Clinical biochemistry						
Leukocytes [per nL], mean (SD)	5.2 (1.97)	6.5 (4.2)	10.4 (8.1)	0.07	<0.0001	0.0001
Hemoglobin [g/dL], mean (SD)	12.2 (1.9)	9.97 (2.4)	8.4 (1.6)	<0.0001	<0.0001	<0.0001
Platelets [per nL], mean (SD)	142.1 (105.6)	128.7 (108.2)	96.5 (65.7)	0.08	0.001	0.1
CRP [mg/dL], mean (SD)	1.0 (1.5)	2.4 (2.5)	4.1 (3.6)	<0.0001	<0.0001	0.001
Sodium [mmol/l], mean (SD)	138.6 (3.3)	135.2 (10.5)	136.6 (6.2)	<0.0001	0.002	0.99
Creatinine [mg/dl], mean (SD)	1.1 (0.9)	1.1 (0.3)	2.4 (1.3)	0.4	<0.0001	<0.0001
Bilirubin [mg/dl], mean (SD)	1.2 (0.6)	4.1 (4.5)	11.3 (11.0)	<0.0001	<0.0001	0.007
AST [U/l], mean (SD)	44.7 (29.7)	68.6 (80.2)	195.9 (896.5)	<0.0001	<0.0001	>0.99
ALT [U/l], mean (SD)	44.8 (38.5)	56.4 (186.7)	83.6 (327.5)	0.2	0.9	>0.99
GGT [U/l], mean (SD)	165.8 (210.8)	133.9 (154.5)	120.7 (162.3)	>0.99	0.2	0.4
AP [U/l], mean (SD)	172.1 (184.5)	166.6 (110.6)	152.1 (112.5)	0.2	>0.99	0.3
INR, mean (SD)	1.2 (0.2)	1.4 (0.3)	2.0 (1.5)	<0.0001	<0.0001	0.002
Albumin [g/dl], mean (SD)	4.4 (2.6)	3.3 (0.6)	3.4 (0.8)	<0.0001	<0.0001	0.5
TSH [mU/l], mean (SD)	2.0 (1.5)	2.9 (2.7)	2.3 (2.9)	0.02	>0.9999	0.02
FT_3_ [pmol/l], mean (SD)	4.8 (0.8)	3.7 (0.8)	3.1 (1.1)	<0.0001	<0.0001	0.0004
FT_4_ [pmol/l], mean (SD)	15.4 (3.3)	15.2 (3.2)	14.8 (4.7)	>0.99	0.6	0.8
ACLF grade						
Grade 1, N (%)	–	–	28 (45.9)	–	–	–
Grade 2, N (%)	–	–	21 (34.4)	–	–	–
Grade 3, N (%)	–	–	12 (19.7)	–	–	–
Complications of liver cirrhosis						
Hepatic encephalopathy						
Grade 0, N (%)	165 (100.0)	172 (82.3)	31 (50.8)	<0.0001	<0.0001	<0.0001
Grade 1, N (%)	0 (0.0)	25 (12.0)	14 (23.0)	<0.0001	<0.0001	0.03
Grade 2, N (%)	0 (0.0)	7 (3.4)	11 (18.0)	0.02	<0.0001	<0.0001
Grade 3, N (%)	0 (0.0)	5 (2.4)	5 (8.2)	0.045	0.0002	0.03
Gastrointestinal bleeding						
N (%)	0 (0.0)	18 (8.5)	9 (14.8)	0.0001	<0.0001	0.2
Infections						
N (%)	12 (7.3)	49 (23.2)	37 (60.7)	<0.0001	<0.0001	<0.0001
Ascites						
No ascites, N (%)	163 (98.8)	71 (33.7)	8 (13.1)	<0.0001	<0.0001	0.002
Moderate, N (%)	2 (1.2)	36 (17.1)	15 (24.6)	<0.0001	<0.0001	0.2
Massive, N (%)	0 (0.0)	104 (49.3)	38 (62.3)	<0.0001	<0.0001	0.07
Esophageal varices						
Grade 0, N (%)	81 (49.1)	73 (34.6)	26 (42.6)	0.005	0.4	0.3
Grade 1, N (%)	36 (21.8)	77 (36.5)	11 (18.0)	0.002	0.5	0.007
Grade 2, N (%)	29 (17.6)	46 (21.8)	21 (34.4)	0.3	0.007	0.04
Grade 3, N (%)	18 (10.9)	14 (6.6)	3 (4.9)	0.1	0.2	0.6
Grade 4, N (%)	1 (0.6)	1 (0.5)	0 (0.0)	0.9	0.5	0.6
Outcome						
Mortality within 90 d, N (%)	3 (1.8)	40 (19.0)	19 (31.15)	<0.0001	<0.0001	0.04
ACLF development within 90 d, N (%)	1 (0.6)	10 (4.7)	–	0.02	–	–

P-values were calculated with One-way ANOVA, Kruskal-Wallis or Chi^2^ -Test, as appropriate.

ALT, alanine aminotransferase; AP, alkaline phosphatase; AST, aspartate aminotransferase; CLIF-OF, chronic liver failure – organ failure; CRP, C-reactive protein; FT_3_, free triiodothyronine; FT_4_, free tetraiodothyronine; GGT, y-glutamyl transferase; INR, international normalized ratio; MELD, model of end-stage liver disease, NASH, non-alcoholic steatohepatitis; TSH, thyroid stimulating hormone.

### 3.2 Evolution of non-thyroidal illness syndrome (NTIS) across clinical stages of liver cirrhosis

**Figure 1:**
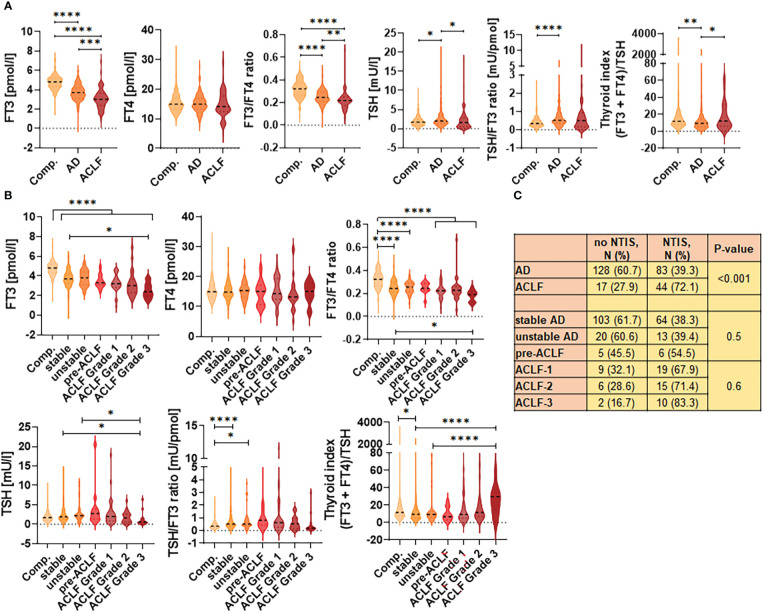
Development of thyroid hormone disbalance during progression of liver cirrhosis. **(A)** Violin blots of thyroid hormone concentrations according to the stage of liver cirrhosis. **(B)** Thyroid hormone concentrations are shown for subgroups of acute decompensation (AD) [stable AD, unstable AD and pre-ACLF], as well as for patients with ACLF grade 1-3. Graphs show value distribution with median (black dashed line) ± quartiles (colored dashed lines). One-way ANOVA or Kruskal-Wallis test were used as appropriate after Normality Test. *P ≤ 0.05, **P ≤ 0.01, ***P ≤ 0.001, ****P≤ 0.0001. **(C)** Frequencies of non-thyroidal illness syndrome (NTIS) according to the stage of liver cirrhosis. ACLF, acute-on-chronic liver failure. AD, acute decompensation; Comp., compensated cirrhosis; FT3, free triiodothyronine; FT4, free tetraiodothyronine; TSH, thyroid-stimulating hormone.

Thyroid hormone levels were quantified at baseline of study inclusion. Concentrations of FT_3_ were significantly lower in patients with AD compared to compensated cirrhosis, and further decreased in patients with ACLF (**Figure 1A**). In contrast, FT_4_ concentrations were not different in patients with compensated cirrhosis, AD and ACLF. As a consequence, a progressive decrease of FT_3_/FT_4_ ratio was observed from compensated cirrhosis to AD and ACLF. Of note, increased TSH concentrations were observed in patients with AD, but not in patients with ACLF, compared to compensated cirrhosis. To obtain more insights into the regulation of the thyroid hormone axis in AD and ACLF, ratios of TSH/FT_3_ as well as the thyroid index ([FT_4_+FT_3_]/TSH) were calculated. Ratios of TSH/FT_3_ were highest in AD and intermediate in ACLF, compared to compensated cirrhosis, while the thyroid index was significantly lower in AD compared to compensated cirrhosis or ACLF. Frequencies of NTIS were 39.3% in AD and 72.1% in ACLF (P<0.001; **Figure 1C**).

Next, AD was further stratified in stable AD, unstable AD and pre-ACLF, and sub-stratification of ACLF in ACLF grade 1-3 was performed (**Figure 1B**). One can note a progressive decrease of FT_3_ concentrations according to the severity of liver disease, while TSH and TSH-based indexes (TSH/FT_3_-ratio, thyroid index) showed a rather inverted U-shaped/U-shaped distribution, suggesting a partially preserved physiological responsiveness of the HPT-axis in patients with AD. NTIS was observed in 38.3% of patients with stable AD, in 39.4% with unstable AD and in 54.5% with pre-ACLF (n.s.), as well as in 67.9%, 71.4% and 83.3% of patients with ACLF grade 1, 2 and 3 (n.s.), respectively (**Figure 1C**). Overall, these changes in thyroid hormones appeared to be independent from the etiology of liver cirrhosis, as shown in **Supplementary Information (SI) Figure 1**.

Collectively, both AD and ACLF are associated with NTIS, which appears to precede ACLF in a proportion of patients, although the TSH secretion from the pituitary in response to low serum T3 concentration is partially maintained in AD.

### 3.3 Impact of infections on NTIS in liver cirrhosis

**Figure 2:**
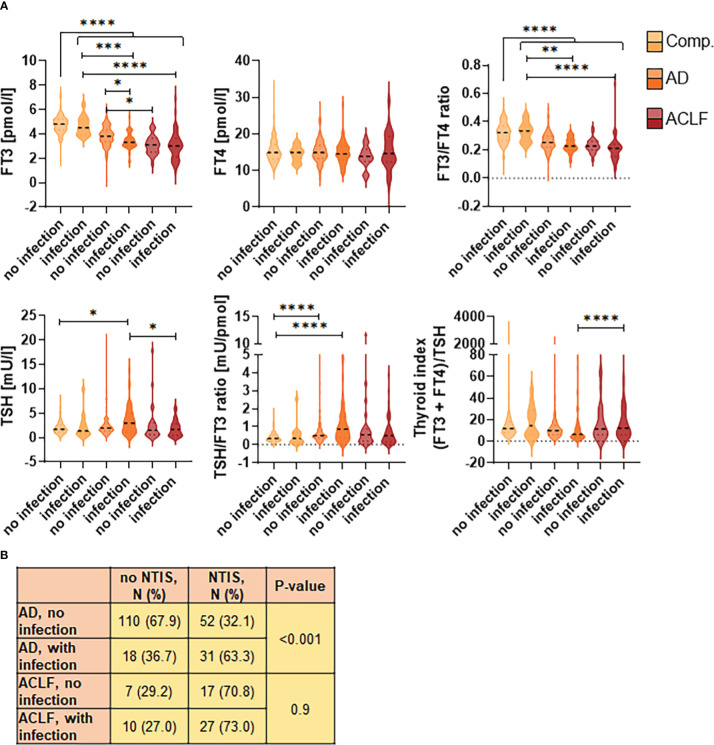
Impact of infections on thyroid hormones. **(A)** Violin blots of thyroid hormone concentrations are shown for patients with or without infections according to the stage of liver cirrhosis. Graphs show value distribution with median (black dashed line) ± quartiles (colored dashed lines). One-way ANOVA or Kruskal-Wallis test were used as appropriate after Normality Test. *P ≤ 0.05, **P ≤ 0.01, ***P ≤ 0.001, ****P≤ 0.0001. **(B)** Frequencies of non-thyroidal illness syndrome (NTIS) in patients with or without infections. ACLF, acute-on-chronic liver failure. AD, acute decompensation; Comp., compensated cirrhosis; FT3, free triiodothyronine; FT4, free tetraiodothyronine; TSH, thyroid-stimulating hormone.

Given the importance of infections as precipitating events and complications of AD and ACLF, we characterized the thyroid hormone axis in patients with or without infections at baseline of study inclusion. In patients with compensated cirrhosis, thyroid hormone concentrations were similar in patients with or without infections (**Figure 2A**). In contrast, patients with AD and infections had significantly lower FT_3_ concentrations (comparable to the concentrations of patients with ACLF) than patients with AD without infections, while the presence of infections in patients with ACLF had no additional effect on thyroid hormone concentrations. Frequencies of NTIS in patients with AD with or without infections were 63.3% vs. 32.1% (P<0.001), and 73.0% vs. 70.8% (n.s.) in patients with ACLF with or without infections (**Figure 2B**). Again, TSH concentrations and TSH-based indices were at least numerically increased (or decreased) in AD with infections, suggest a certain responsiveness of the thyroid gland in these patients.

### 3.4 Association between NTIS and mortality, clinical scores and hepatic encephalopathy

**Figure 3:**
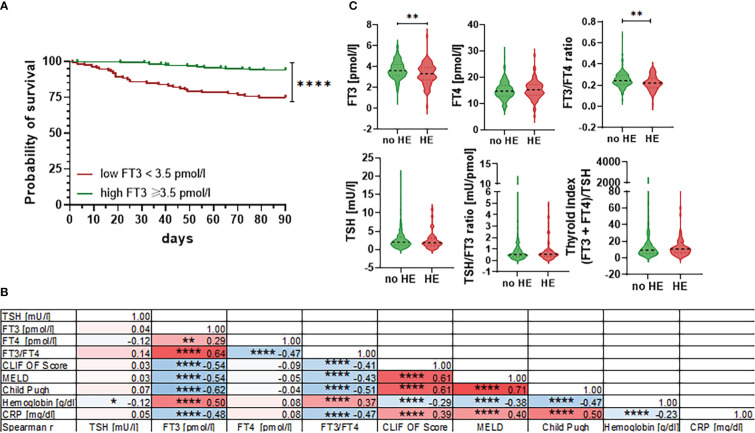
Association between thyroid hormones and 90-day mortality, clinical scores and hepatic encephalopathy. **(A)** Probability of 90-day survival in patients with FT3 concentrations <3.5 pmol/L versus ≥3.5 pmol/L. Log-rank test P<0.0001. **(B)** Correlation matrix of selected thyroid hormone values, clinical scores and laboratory values of patients showing spearman r values and levels of significance. CRP, C-reactive protein; CLIF OF, CLIF organ failure score; FT3, free triiodothyronine; FT4, free tetraiodothyronine; MELD, Model for End-Stage Liver Disease; TSH, thyroid-stimulating hormone. *P ≤ 0.05, **P ≤ 0.01, ***P ≤ 0.001, ****P≤ 0.0001. **(C)** Violin blots of thyroid hormone concentrations are shown for patients with or without hepatic encephalopathy (HE). Graphs show value distribution with median (black dashed line) ± quartiles (colored dashed lines). One-way ANOVA or Kruskal-Wallis test were used as appropriate after Normality Test. *P ≤ 0.05, **P ≤ 0.01, ***P ≤ 0.001, ****P≤ 0.0001.

Next, we assessed associations between baseline thyroid hormone concentrations and transplant-free survival within 90 days. As shown in **Figure 3A**, FT_3_ concentration below the lower limit of normal was significantly associated with the risk of death within 90 days. In multivariate Cox regression analysis, FT_3_ concentrations remained an independent predictor of 90-day mortality (P=0.002; OR 0.71 (95% CI 0.47-0.91)), together with age (P=0.003; OR 1.04 (95% CI 1.01-1.08)), the MELD score (P=0.0005; OR 1.06 (95% CI 1.01-1.13)), and leukocytes (P=0.004; OR 1.10 (95% CI 1.03-1.18)), **Table 2**. Furthermore, low FT_3_ concentrations (and the FT_3_/FT_4_ ratio) correlated significantly with the MELD-, CLIF-OF-, and Child-Pugh-Score, as well as with C-reactive protein and low haemoglobin concentrations (**Figure 3B**). In multivariate analysis, the CLIF OF score (P=0.01; OR 1.51 (95% CI 1.11-2.11)), CRP (P=0.03; OR 1.21 (95% CI 1.03-1.45)) and serum albumin (P=0.002; OR 0.36 (95% CI 0.19-0.67)) were independent predictors of the presence of NTIS (**Table 3**).

**Table 2:** Cox-regression analysis of 90-day mortality.

Mortality within 90 days	Univariate analysis		Multivariate analysis	
	OR (95% CI)	P value	OR (95% CI)	P value
Age [years], continuous	1.04 (1.02-1.07)	0.002	1.04 (1.01-1.08)	0.003
Gender, male vs. female	1.50 (0.84-2.69)	0.2		
CLIF OF score, continuous	1.54 (1.34-1.78)	<0.0001		
MELD, continuous	1.12 (1.08-1.17)	<0.0001	1.06 (1.01-1.13)	0.0005
Leukocytes [/nL], continuous	1.14 (1.08-1.21)	<0.0001	1.10 (1.03-1.18)	0.004
Hemoglobin [g/dL], continuous	0.76 (0.66-0.86)	<0.0001		
FT3 [pmol/L], continuous	0.39 (0.28-0.53)	<0.0001	0.71 (0.47-0.91)	0.002
Presence of hepatic encephalopathy, (yes/no)	4.16 (2.16-7.91)	<0.0001		
Presence of infection, (yes/no)	3.54 (1.93-6.47)	<0.0001		
Presence of ascites, (yes/no)	7.55 (3.63-17.8)	<0.0001		

**Table 3:** Regression analysis of factors associated with non-thyroidal illness syndrome (NTIS).

	Univariate analysis		Multivariate analysis	
	OR (95% CI)	P value	OR (95% CI)	P value
CLIF OF score, continuous	1.83 (1.59-2.14)	<0.0001	1.51 (1.11-2.11)	0.01
MELD score, continuous	1.14 (1.10-1.18)	<0.0001		
Leukocytes [/nL], continuous	1.10 (1.05-1.15)	0.0002		
Hemoglobin [g/dL], continuous	0.61 (0.55-0.68)	<0.0001		
Platelets [/nL], continuous	0.99 (0.99-1.00)	0.01		
CRP [mg/dL], continuous	1.46 (1.31-1.64)	<0.0001	1.21 (1.03-1.45)	0.03
Albumin [g/dL], continuous	0.22 (0.13-0.33)	<0.0001	0.36 (0.19-0.67)	0.002
Presence of hepatic encephalopathy, (yes/no)	3.40 (1.99-5.83)	<0.0001		
Presence of infection, (yes/no)	5.06 (3.15-8.21)	<0.0001		
Presence of ascites, (yes/no)	11.99 (7.18-20.82)	<0.0001		

Since thyroxin concentrations were associated with hepatic encephalopathy (HE) in a recent metabolome study (16), we further assessed association between thyroid hormone concentrations and presence vs. absence of HE. Indeed, patients with HE had lower FT_3_ concentrations and lower FT_3_/FT_4_ ratios compared to patients without HE, while TSH concentrations and TSH-based indices were similar in patients with versus without HE (**Figure 3C**).

### 3.5 Relationship between NTIS, stage of liver cirrhosis and systemic inflammation

Since systemic inflammation is a hallmark of AD and ACLF, but also of NTIS, we assessed the relationship between NTIS, stage of liver cirrhosis and systemic inflammation. To this end, 76 plasma proteins and inflammatory mediators were analysed in a subgroup of 104 patients with available plasma by proximity extension analysis assay. Baseline characteristics of this subgroup are shown in **SI Table 1** and are comparable to the entire cohort.

**Figure 4:**
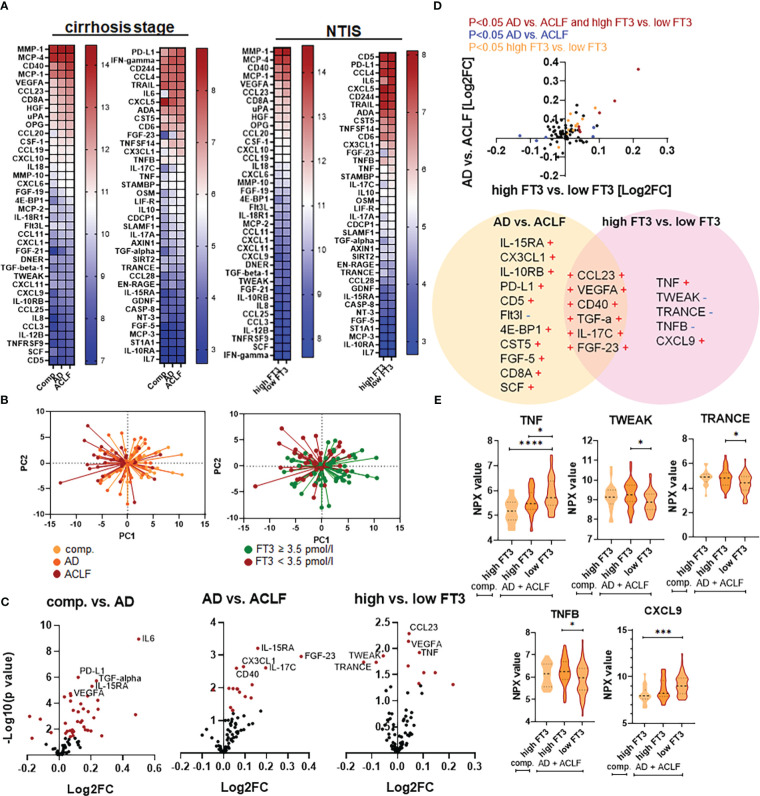
Patterns of systemic inflammation according to the stage of cirrhosis and according to the presence/absence of non-thyroidal illness syndrome (NTIS). **(A)** Plasma proteins and inflammatory mediators were quantified by proximity extension analysis assay. Heat-maps of 76 proteins that passed the quality controls are shown according to the stage of cirrhosis (left two panels) and according to the presence/absence of NTIS (right two panels). **(B)** Principal component analysis of plasma proteins and inflammatory mediators are shown of patients with compensated cirrhosis (comp.), acute decompensation (AD), or acute-on-chronic liver failure (ACLF) (left panel) and of patients with AD and ACLF with versus without NTIS (right panel). **(C)** Volcano blots of plasma proteins and mediators differentially regulated (dots in red mark differentially regulated, i.e. statistically different mediators) in compensated cirrhosis vs. AD, in AD vs. ACLF, and in patients with FT3 concentrations <3.5 pmol/L vs. ≥3.5 pmol/L. **(D)** Plasma proteins and mediators differentially regulated in patients with AD and ACLF only, excluding patients with compensated liver cirrhosis. A volcano blot showing comparisons between patients with AD and ACLF vs. patients with FT3 concentrations <3.5 pmol/L vs. ≥3.5 pmol/L is shown in the upper row. Names of the significantly changed proteins are depicted in the lower row. **(E)** Violin blots of plasma proteins which are significantly changed in patients with FT3 concentrations <3.5 pmol/L vs. ≥3.5 pmol/L exclusively, but not in AD vs. ACLF. Graphs show value distribution with median (black dashed line) ± quartiles (colored dashed lines). One-way ANOVA or Kruskal-Wallis test were used as appropriate after Normality Test. *P ≤ 0.05, ***P ≤ 0.001, ****P≤ 0.0001.

As expected, AD and in particular ACLF were associated with significant changes in plasma proteins and inflammatory mediators (**Figures 4A, B**). In detail, 35 and 4 proteins were significantly up- and down-regulated in patients with AD compared to patients with compensated cirrhosis, respectively (**Figure 4C** and **SI Table 2**), while 16 and 1 proteins were significantly up- and down-regulated in patients with ACLF compared to patients with AD, respectively (**Figure 4C** and **SI Table 3**). Further analysis showed a clear association between NTIS and systemic inflammation as well (**Figures 4A-D**). Overall, 8 and 3 proteins were significantly up- and down-regulated in patients with NTIS compared to patients without NTIS, respectively (**Figure 4C** and **SI Table 4**).

**Figure 5:**
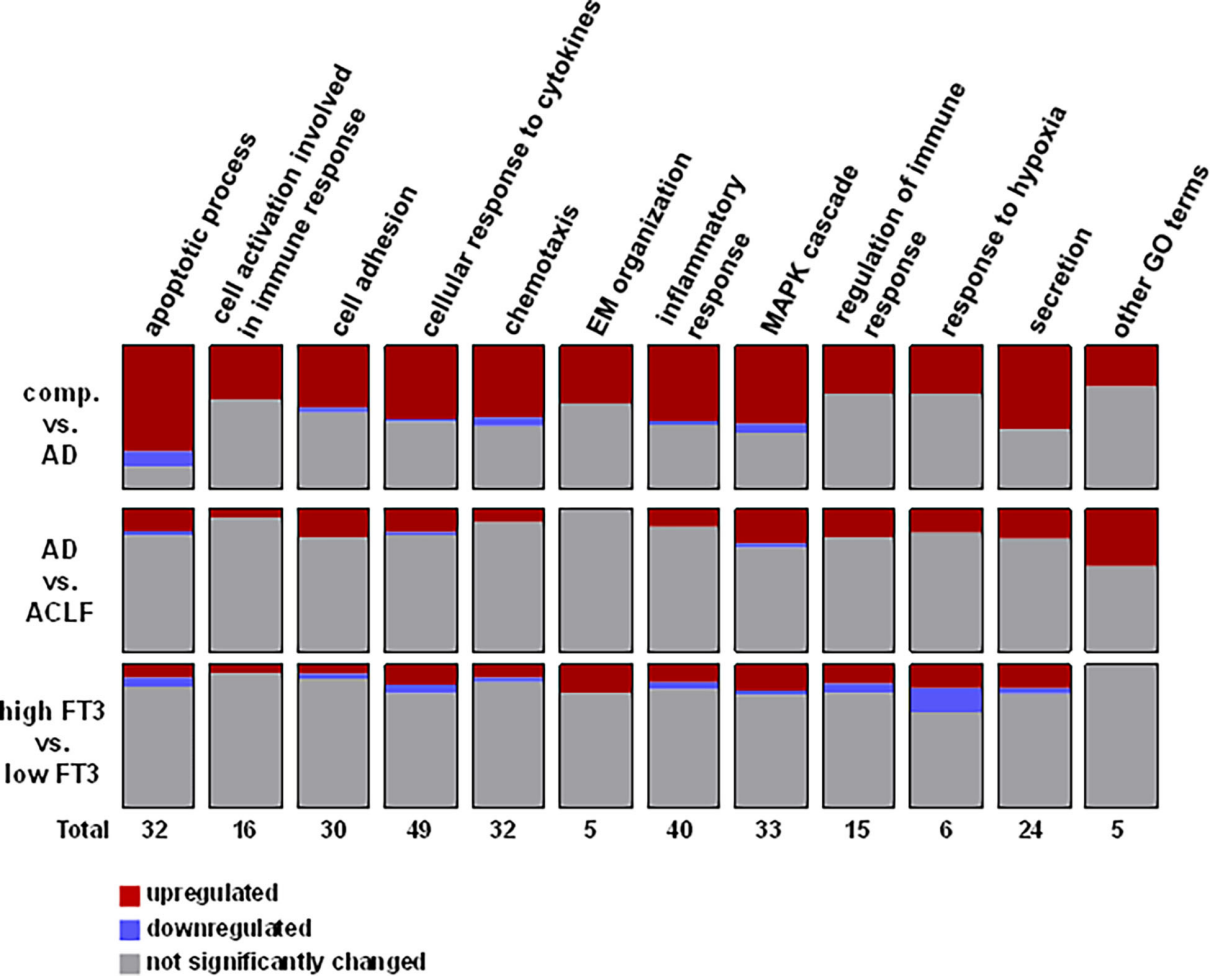
Pathway analysis of proteins detected by proximity extension analysis assay.

Since AD/ACLF and NTIS are associated clinical conditions, we next examined whether NTIS-associated changes in plasma proteins and inflammatory mediators are partially independent from the signatures of AD and ACLF. To this end, plasma proteins were analysed in the subgroup of patients with AD and ACLF with or without NTIS, excluding patients with compensated cirrhosis. As shown in **Figure 4D**, a number of 6 plasma proteins and inflammatory mediators were significantly upregulated in ACLF vs. AD and in patients with or without NTIS, while 15 and 6 plasma proteins where significantly regulated in ACLF vs. AD or in patients with or without NTIS only. Proteins that were significantly regulated in NTIS only included the members of the TNF-superfamily TNF-α, TWEAK, TRANCE, and TNF-β (**Figure 4E**). A further pathway analysis showed some global differences between pathways regulated in AD/ACLF versus NTIS, as for example more proteins involved in response to hypoxia were downregulated in NTIS compared to AD or ACLF (**Figure 5**).

## 4 Discussion

In the present study we provide a comprehensive analysis of thyroid hormones across the re-defined clinical stages of decompensated liver cirrhosis according to the results of the CANONIC- and PREDICT-studies (2, 5). The main findings of our study are that i) NTIS is frequent in both, patients with AD and ACLF, ii) NTIS is a strong predictor of 90-day mortality, and iii) that NTIS and AD/ACLF are associated with partially non-overlapping systemic inflammation patterns.

NTIS is a well-known sequalae of critical illness conditions like sepsis, trauma or cardiac surgery (9). In such clinical scenarios, thyroid hormone levels are reduced by several mechanisms including a reduced hypothalamic and pituitary secretion of TRH and TSH and a reduced conversion of FT_4_ to FT_3_ (9). Mechanistically, inflammatory mediators like IL-6 contribute to the pathogenesis of NTIS by inhibiting hormone secretion and conversion. For example, a study has shown that generation of the active T_3_ by DIO1 and DIO2 is inhibited by IL-6, which – interestingly – could be prevented by N-acetyl-cysteine-induced glutathione restoration (17). Of note, hepatic DIO1 is a key converting enzyme required for efficient conversion of FT_4_ to FT_3_, and it is known that hepatic DIO1 expression is reduced in acute non-hepatic diseases (8). Hence, in liver cirrhosis, a reduced DIO1 capacity in the liver (and perhaps depleted glutathione levels), might be additional mechanisms to systemic inflammation of NTIS in AD and ACLF, which may explain the relatively early occurrence of NTIS before patients enter a critically ill state. In line with this, our observation of an increase in TSH-concentrations and TSH-based indices in AD compared to compensated cirrhosis and ACLF suggests an impaired hepatic conversion to FT_3_ already in AD, while inflammatory mechanisms causing NTIS in this stage are not yet fully developed. In contrast, the full-blown systemic inflammation in ACLF leads to inability to increase TSH in response to low FT_3_ levels – a major pathophysiological feature of NTIS – resulting in fully developed features of NTIS in ACLF (9).

An impact of low thyroid hormones in patients with liver cirrhosis have been reported in previous studies. For example, studies have described low FT_3_ levels in critically ill patients with cirrhosis, an increase of thyroid hormone dysfunction according to Child Pugh-stage, or the need of increased doses of T_4_ to maintain optimal FT_3_ concentrations according to the severity of liver disease (15, 18–20). Most recently, an untargeted metabolome analysis has identified low thyroxine concentrations in hospitalized patients with hepatic encephalopathy (16). In this multicentre study of 602 patients, of whom 144 developed hepatic encephalopathy within three days of admission, untargeted serum metabolomics revealed that low thyroxine at baseline was an independent predictor of brain failure development. More in general, profound differences in the blood metabolome of patients with AD versus ACLF have also been shown by Moreau et al. in a large study, suggesting links between systemic inflammation, metabolomics and mitochondrial dysfunction (5). Yet, to our knowledge, we provide for the first detailed analysis of the evolution of disturbances of the thyroid hormone axis according to the recent re-classification of decompensated cirrhosis derived from the results of the CANONIC- and PREDICT-studies. These recent definitions have, in our view, significantly improved the classification of AD and ACLF, although the concept of ACLF per se is not new.

At present, we can only speculate about the clinical implications of our findings. The important question is whether low T_3_ is a detrimental consequence of inflammation in AD and ACLF that should be corrected with TH substitution or is a response that protects the patient from hypermetabolism in a fragile situation and should not be corrected. Clinical data on the value of thyroid hormone replacement therapy in critically ill patients are scarce and partially conflicting, but have overall failed to demonstrate a benefit (9). However, the clinical course of AD and ACLF is distinct from syndromes like sepsis, as the latter develops within hours in a previously healthy organism, while ACLF-defining organ failures can develop in a prolonged manner on the basis of preexisting and usually decompensated liver cirrhosis. In this regard, the recent PREDICT-study has revealed that patients with AD (namely unstable AD or pre-ACLF) are at high risk of developing adverse events like infections, ACLF or death within a time-frame of 3 months (2). On a pathophysiological basis, a beneficial effect of thyroid hormone action in the prevention of adverse outcomes in patients with AD and ACLF appears plausible, since thyroid hormone signaling is required for immune cell homeostasis and appropriate mitochondrial function (9). Furthermore, TH promotes hepatocyte proliferation and liver regeneration (21). These are all important determinants in the development and outcome of ACLF. In this regard, our observation that NTIS is a feature of AD, preceding ACLF, may provide a therapeutic window to apply thyroid hormone action in a preventive manner, i.e. before the occurrence of ACLF and death. In this regard, a recent meta-analysis showed that thyroid hormone treatment was effective in patients with heart failure suffering from NTIS (22).Therefore, future studies are justified to explore the clinical value of thyroid hormone therapy or, possibly, therapy with liver-specific TRβ-agonists in order to harvest beneficial hepatic effects while avoiding adverse extrahepatic effects in AD and ACLF.

Systemic inflammation is a hallmark of both AD/ACLF and of NTIS (3, 9, 10), although it is not clear whether systemic inflammation represents an alteration of the host response to injury or an inability to resolve inflammation (23). In our study, we have replicated the known association with systemic inflammation and AD/ACLF by quantifying a large number of plasma proteins and mediators involved in inflammation and immune responses. As expected, the magnitude of systemic inflammation was highest in patients with ACLF and intermediate in patients with AD, compared to compensated cirrhosis. In addition, we could show significant differences in markers of systemic inflammation with versus without NTIS. Since NTIS and AD/ACLF are associated with each other, this observation is not surprising. However, the signatures of inflammatory and immune plasma proteins and mediators of AD/ACLF and NITS were only partially overlapping. For example, a number of proteins of the TNF-superfamily (e.g. TWEAK, TRANCE) were significantly regulated only in NTIS, but not in AD/ACLF. Collectively, our data suggest that NTIS is a feature of AD and ACLF, and probably partially induced by AD and ACLF-associated inflammatory mediators, but NTIS may in turn modulate the immune and inflammatory response in patients with decompensated cirrhosis.

Our study has limitations. Most importantly, the study design is cross-sectional, and we have collected only baseline but not repetitive longitudinal data of thyroid hormones of patients who progress to other cirrhosis stages (e.g. from AD to ACLF).

In conclusion, NTIS is frequent not only in ACLF but also frequent already in patients with AD, and appears to precede the development of critical illness in decompensated cirrhosis. This might offer chances to explore a possible therapeutic role of thyroid hormone action to prevent disease progression in AD and early ACLF.

## Data availability statement

The original contributions presented in the study are included in the article/**Supplementary Material**. Further inquiries can be directed to the corresponding author.

## Ethics statement

The studies involving human participants were reviewed and approved by local ethical committee. The patients/participants provided their written informed consent to participate in this study.

## Author contributions

M-ML, CL: Study design, sample collection, data acquisition, statistical analysis, interpretation of data, manuscript writing. AB, SG, SK, TL, GD, DZ, LM: Data acquisition, interpretation of data, manuscript editing. All authors contributed to the article and approved the submitted version.
