# Supplementary material for: Evolution of non-thyroidal illness syndrome in acute decompensation of liver cirrhosis and acute-on-chronic liver failure
Source: Front Endocrinol (Lausanne). 2023 Jan 23;14:1104388. doi: 10.3389/fendo.2023.1104388 (PMC9899974; doi:10.3389/fendo.2023.1104388)
Supplement: Supplementary file 1 [file DataSheet_1.docx]

**Supplementary Information**

**SI table 1. Baseline characteristics and laboratory results of patients in sub cohort.**

|  | Comp. (N=20) | AD (N=52) | ACLF (N=32) | P-value  (Comp. vs. AD) | P-value  (Comp. vs. ACLF) | P-value  (AD vs. ACLF) |
| --- | --- | --- | --- | --- | --- | --- |
|  |  |  |  |  |  |  |
| General characteristics | | | | | | |
| Age [years], mean (SD) | 48.2 (13.4) | 60.2 (12.1) | 57.0 (10.2) | 0.0006 | 0.02 | 0.2 |
| Male gender; female gender, N (%) | 11 (55.0); 9 (45.0) | 34 (65.4); 18 (34.6) | 22 (68.8); 10 (31.3) | 0.4 | 0.3 | 0.8 |
| Child Pugh Score, mean (SD) | 5.3 (0.5) | 8.4 (1.3) | 9.3 (1.6) | <0.0001 | <0.0001 | 0.1 |
| CLIF OF score, mean (SD) | 6.6 (1.1) | 7.0 (1.1) | 10.0 (2.0) | 0.6 | <0.0001 | <0.0001 |
| MELD score, mean (SD) | 7.7 (2.8) | 13.8 (5.5) | 26.6 (9.2) | 0.002 | <0.0001 | <0.0001 |
| Etiology of liver cirrhosis | | | | | | |
| Viral, N (%) | 1 (5.0) | 4 (7.7) | 2 (6.3) | 0.7 | 0.9 | 0.8 |
| NASH, N (%) | 3 (15.0) | 6 (11.5) | 5 (15.6) | 0.7 | 0.96 | 0.6 |
| Alcoholic, N (%) | 9 (45.0) | 28 (53.9) | 24 (75.0) | 0.5 | 0.03 | 0.053 |
| Cholestatic, N (%) | 4 (20.0) | 2 (3.9) | 0 (0.0) | 0.03 | 0.009 | 0.3 |
| Others, N (%) | 3 (15.0) | 12 (23.1) | 1 (3.1) | 0.4 | 0.1 | 0.01 |
| Clinical biochemistry | | | | | | |
| Leukocytes [per nL], mean (SD) | 5.4 (1.9) | 8.0 (5.3) | 9.6 (6.9) | 0.07 | 0.03 | >0.99 |
| Hemoglobin [g/dL], mean (SD) | 11.7 (2.3) | 9.3 (2.4) | 8.8 (1.9) | 0.0001 | <0.0001 | 0.3 |
| Platelets [per nL], mean (SD) | 151.3 (75.9) | 156.9 (106.4) | 112.8 (62.7) | >0.99 | 0.2 | 0.2 |
| CRP [mg/dL], mean (SD) | 0.7 (0.5) | 3.2 (3.2) | 3.8 (3.8) | <0.0001 | <0.0001 | 0.8 |
| Sodium [mmol/l], mean (SD) | 138.3 (2.9) | 133.5 (5.9) | 135.8 (5.7) | 0.002 | 0.07 | 0.7 |
| Creatinine [mg/dl], mean (SD) | 0.9 (0.2) | 1.2 (0.4) | 2.6 (1.2) | 0.3 | <0.0001 | <0.0001 |
| Bilirubin [mg/dl], mean (SD) | 1.2 (0.7) | 3.4 (4.1) | 11.1 (12.5) | 0.03 | 0.0001 | 0.1 |
| AST [U/l], mean (SD) | 49.1 (40.0) | 63.9 (70.5) | 83.3 (86.2) | 0.3 | 0.3 | >0.99 |
| ALT [U/l], mean (SD) | 41.2 (35.5) | 35.1 (27.1) | 45.1 (44.1) | >0.99 | >0.99 | >0.99 |
| GGT [U/l], mean (SD) | 187.7 (233.0) | 161.2 (149.0) | 152.6 (204.7) | >0.99 | >0.99 | 0.6 |
| AP [U/l], mean (SD) | 184.1 (168.6) | 170.9 (92.1) | 153.6 (109.9) | >0.99 | >0.99 | 0.4 |
| INR, mean (SD) | 1.1 (0.1) | 1.4 (0.3) | 2.1 (1.7) | 0.001 | <0.0001 | 0.01 |
| Albumin [g/dl], mean (SD) | 4.3 (0.5) | 3.2 (0.6) | 3.2 (0.7) | <0.0001 | <0.0001 | 0.9 |
| TSH [mU/l], mean (SD) | 2.7 (2.1) | 3.5 (4.0) | 2.2 (2.2) | >0.99 | 0.9 | 0.3 |
| FT_3_ [pmol/l], mean (SD) | 4.9 (0.6) | 3.6 (0.7) | 3.4 (1.0) | <0.0001 | <0.0001 | 0.5 |
| FT_4_ [pmol/l], mean (SD) | 15.6 (4.7) | 15.2 (2.8) | 14.8 (4.6) | >0.99 | >0.99 | 0.7 |
| ACLF grade | | | | | | |
| Grade 1, N (%) | - | - | 16 (50.0) | - | - | - |
| Grade 2, N (%) | - | - | 13 (40.1) | - | - | - |
| Grade 3, N (%) | - | - | 3 (9.4) | - | - | - |
| Complications of liver cirrhosis | | | | | | |
| *Hepatic encephalopathy* | | | | | | |
| Grade 0, N (%) | 20 (0.0) | 41 (78.9) | 17 (53.1) | 0.03 | 0.0003 | 0.01 |
| Grade 1, N (%) | 0 (0.0) | 8 (15.4) | 10 (31.3) | 0.06 | 0.005 | 0.09 |
| Grade 2, N (%) | 0 (0.0) | 1 (1.9) | 4 (12.5) | 0.5 | 0.1 | 0.047 |
| Grade 3, N (%) | 0 (0.0) | 2 (3.9) | 1 (3.1) | 0.4 | 0.4 | 0.9 |
| *Gastrointestinal bleeding* | | | | | | |
| N (%) | 0 (0.0) | 6 (11.5) | 3 (9.4) | 0.1 | 0.2 | 0.8 |
| *Infections* | | | | | | |
| N (%) | 0 (0.0) | 19 (36.5) | 17 (53.1) | 0.002 | <0.0001 | 0.1 |
| *Ascites* | | | | | | |
| No ascites, N (%) | 20 (100.0) | 6 (11.5) | 3 (9.4) | <0.0001 | <0.0001 | 0.8 |
| Moderate, N (%) | 0 (0.0) | 20 (38.5) | 8 (25.0) | 0.001 | 0.02 | 0.2 |
| Massive, N (%) | 0 (0.0) | 26 (50.0) | 21 (65.6) | <0.0001 | <0.0001 | 0.2 |
| *Esophageal varices* | | | | | | |
| Grade 0, N (%) | 11 (55.0) | 17 (32.7) | 12 (37.5) | 0.08 | 0.2 | 0.7 |
| Grade 1, N (%) | 2 (10.0) | 19 (36.5) | 6 (18.8) | 0.03 | 0.4 | 0.08 |
| Grade 2, N (%) | 5 (25.0) | 10 (19.2) | 13 (40.6) | 0.6 | 0.2 | 0.03 |
| Grade 3, N (%) | 2 (10.0) | 6 (11.5) | 1 (3.1) | 0.9 | 0.3 | 0.2 |
| Outcome | | | | | | |
| Mortality within 90 d, N (%) | 0 (0.0) | 11 (21.2) | 8 (25.0) | 0.03 | 0.02 | 0.7 |
| ACLF development within 90 d, N (%) | 0 (0.0) | 7 (13.5) | - | 0.08 | - | - |

**SI Table 2. Number of proteins detected by Olink in compensated liver cirrhosis vs. AD.**

| **Target** | **comp.** | **AD** | **p value** |
| --- | --- | --- | --- |
|  | (median, IQR, SEM  [NPX value]) | (median, IQR, SEM  [NPX value])2 |  |
| **CCL23** | 11.58, 0.77, 1.10 | 12.04, 0.72, 0.08 | **P<0.0001** |
| **CD40** | 12.42, 0.78, 0.12 | 13.02, 0.65, 0.07 | **P<0.0001** |
| **CSF-1** | 10.97, 0.24, 0.05 | 11.26, 0.23, 0.02 | **P<0.0001** |
| **CX3CL1** | 5.43, 0.70, 0.12 | 6.12, 0.76, 0.09 | **P<0.0001** |
| **IL-15RA** | 3.26, 0.45, 0.07 | 3.71, 0.80, 0.07 | **P<0.0001** |
| **IL6** | 5.30, 0.74, 0.23 | 7.55, 1.37, 0.17 | **P<0.0001** |
| **IL8** | 6.84, 1.38, 0.22 | 8.14, 1.69, 0.18 | **P<0.0001** |
| **PD-L1** | 6.97, 0.58, 0.08 | 7.43, 0.64, 0.06 | **P<0.0001** |
| **TGF-α** | 3.88, 0.70, 0.10 | 4.52, 0.85, 0.08 | **P<0.0001** |
| **VEGFA** | 11.59, 0.68, 0.10 | 12.08, 0.52, 0.07 | **P<0.0001** |
| **CCL20** | 10.16, 1.48, 0.24 | 11.22, 1.71, 0.15 | **P<0.001** |
| **CCL25** | 8.09, 0.62, 0.10 | 8.59, 0.61, 0.08 | **P<0.001** |
| **CCL3** | 7.36, 0.59, 0.12 | 7.85, 1.09, 0.13 | **P<0.001** |
| **FGF-23** | 3.31, 2.45, 0.33 | 5.21, 2.30, 0.22 | **P<0.001** |
| **FGF-5** | 2.79, 0.33, 0.06 | 3.06, 0.30, 0.08 | **P<0.001** |
| **IL-17C** | 4.22, 0.59, 0.15 | 4.73, 1.53, 0.15 | **P<0.001** |
| **TNFSF14** | 6.03, 0.76, 0.11 | 6.69, 0.78, 0.08 | **P<0.001** |
| **4E-BP1** | 8.60, 1.51, 0.23 | 9.50, 1.36, 0.13 | **P<0.01** |
| **CXCL9** | 7.96, 0.97, 0.20 | 8.68, 2.05, 0.16 | **P<0.01** |
| **IL-10RB** | 8.57, 0.36, 0.07 | 8.77, 0.34, 0.03 | **P<0.01** |
| **IL-17A** | 4.23, 0.95, 0.17 | 5.11, 0.92, 0.12 | **P<0.01** |
| **NT-3** | 3.92, 0.51, 0.12 | 3.45, 0.43, 0.06 | **P<0.01** |
| **SCF** | 8.69, 1.53, 0.18 | 8.02, 0.66, 0.06 | **P<0.01** |
| **SLAMF1** | 4.60, 1.12, 0.16 | 5.13, 0.59, 0.08 | **P<0.01** |
| **TNF** | 5.19, 0.72, 0.11 | 5.51, 0.75, 0.08 | **P<0.01** |
| **CCL11** | 9.07, 1.01, 0.15 | 9.70, 1.06, 0.10 | **P<0.05** |
| **CDCP1** | 4.85, 1.08, 0.19 | 5.21, 0.80, 0.09 | **P<0.05** |
| **CXCL5** | 9.45, 3.72, 0.43 | 7.85, 2.20, 0.22 | **P<0.05** |
| **CXCL6** | 10.25, 1.25, 0.18 | 10.49, 1.29, 0.13 | **P<0.05** |
| **FGF-19** | 9.47, 1.19, 0.20 | 10.22, 1.57, 0.19 | **P<0.05** |
| **FGF-21** | 6.81, 4.03, 0.55 | 8.62, 3.98, 0.32 | **P<0.05** |
| **HGF** | 11.22, 1.30, 0.22 | 11.82, 1.30, 0.13 | **P<0.05** |
| **IL10** | 4.75, 0.80, 0.21 | 5.32, 0.96, 0.19 | **P<0.05** |
| **LIF-R** | 5.08, 0.75, 0.11 | 5.38, 0.57, 0.06 | **P<0.05** |
| **OPG** | 11.30, 0.60, 0.12 | 11.54, 0.64, 0.06 | **P<0.05** |
| **OSM** | 4.61, 1.36, 0.19 | 5.05, 1.09, 0.14 | **P<0.05** |
| **TNFRSF9** | 7.54, 0.98, 0.14 | 7.81, 0.76, 0.11 | **P<0.05** |
| **TRAIL** | 7.91, 0.42, 0.10 | 7.61, 0.54, 0.05 | **P<0.05** |
| **uPA** | 11.44, 1.19, 0.14 | 11.88, 0.74, 0.07 | **P<0.05** |
| ADA | 7.03, 0.38, 0.09 | 7.09, 0.80, 0.10 | ns |
| AXIN1 | 4.66, 1.82, 0.30 | 4.95, 1.49, 014 | ns |
| CASP-8 | 3.25, 1.44, 0.27 | 3.67, 0.90, 0.13 | ns |
| CCL19 | 10.82, 1.31, 0.20 | 11.13, 1.54, 0.14 | ns |
| CCL28 | 3.85, 0.88, 0.18 | 4.36, 0.67, 0.08 | ns |
| CCL4 | 7.34, 0.94, 0.16 | 7.36, 0.71, 0.15 | ns |
| CD244 | 7.41, 0.93, 0.11 | 7.52, 0.90, 0.08 | ns |
| CD5 | 7.48, 0.58, 0.11 | 7.74, 0.68, 0.06 | ns |
| CD6 | 6.85, 1.13, 0.16 | 6.77, 0.98, 0.10 | ns |
| CD8A | 11.44, 1.38, 0.22 | 11.95, 0.97, 0.13 | ns |
| CST5 | 6.31, 0.83, 0.15 | 6.67, 1.19, 0.11 | ns |
| CXCL1 | 9.02, 1.71, 0.24 | 9.41, 1.13, 0.15 | ns |
| CXCL10 | 10.65, 2.16, 0.27 | 11.09, 1.58, 0.15 | ns |
| CXCL11 | 9.14, 1.02, 0.25 | 9.09, 1.51, 0.14 | ns |
| DNER | 9.22, 0.33, 0.05 | 9.17, 0.38, 0.06 | ns |
| EN-RAGE | 3.87, 0.88, 0.28 | 4.09, 2.05, 0.20 | ns |
| Flt3L | 10.09, 0.39, 0.09 | 10.17, 0.63, 0.08 | ns |
| GDNF | 3.42, 0.8, 0.6 | 3.81, 0.91, 0.09 | ns |
| IFN-ɣ | 7.20, 1.19, 0.18 | 7.74, 2.10, 0.19 | ns |
| IL-10RA | 2.25, 0.48, 0.23 | 2.67, 0.52, 0.06 | ns |
| IL-12B | 8.26, 0.99, 0.15 | 8.20, 1.50, 0.14 | ns |
| IL18 | 9.96, 1.08, 0.21 | 10.48, 0.98, 0.14 | ns |
| IL-18R1 | 9.34, 0.87, 0.18 | 9.67, 0.70, 0.07 | ns |
| IL7 | 2.96, 0.76, 0.12 | 2.68, 0.58, 0.08 | ns |
| LAP TGF-β-1 | 8.84, 0.59, 0.11 | 9.02, 0.53, 0.06 | ns |
| MCP-1 | 12.75, 0.90, 0.12 | 12.95, 0.85, 0.09 | ns |
| MCP-2 | 9.83, 0.57, 0.15 | 9.87, 1.08, 0.10 | ns |
| MCP-3 | 2.55, 0.66, 0.15 | 2.89, 0.55, 0.08 | ns |
| MCP-4 | 13.68, 1.71, 0.23 | 1.372, 1.20, 0.13 | ns |
| MMP-1 | 13.66, 1.89, 0.28 | 14.55, 1.22, 0.13 | ns |
| MMP-10 | 10.20, 0.85, 0.18 | 10.63, 1.25, 0.11 | ns |
| SIRT2 | 4.75, 1.78, 0.25 | 4.60, 0.99, 0.14 | ns |
| ST1A1 | 3.13, 1.41, 0.19 | 3.12, 1.02, 0.12 | ns |
| STAMBP | 5.47, 1.04, 0.20 | 5.53, 0.83, 0.11 | ns |
| TNFB | 6.16, 1.02, 0.12 | 6.12, 0.83, 0.09 | ns |
| TRANCE | 4.90, 0.61, 0.14 | 4.52, 1.17, 0.12 | ns |
| TWEAK | 9.14, 0.71, 0.15 | 9.03, 1.16, 0.10 | ns |

**SI Table 3. Number of proteins detected by Olink in AD vs. ACLF.**

| **Target** | **AD** | **ACLF** | **p value** |
| --- | --- | --- | --- |
|  | (median, IQR, SEM  [NPX value]) | (median, IQR, SEM  [NPX value])2 |  |
| **IL-15RA** | 3.71, 0.80, 0.07 | 4.25, 0.55, 0.10 | **P<0.001** |
| **CD40** | 13.02, 0.65, 0.07 | 13.57, 1.08, 0.16 | **P<0.01** |
| **CX3CL1** | 6.12, 0.76, 0.09 | 6.54, 0.82, 0.09 | **P<0.01** |
| **FGF-23** | 5.21, 2.30, 0.22 | 6.89, 2.79, 0.36 | **P<0.01** |
| **IL-17C** | 4.73, 1.53, 0.15 | 5.90, 1.58, 0.18 | **P<0.01** |
| **TGF-α** | 4.52, 0.85, 0.08 | 4.92, 0.74, 0.14 | **P<0.01** |
| **4E-BP1** | 9.50, 1.36, 0.13 | 10.14, 1.40, 0.16 | **P<0.05** |
| **CCL23** | 12.04, 0.72, 0.08 | 12.40, 0.62, 0.10 | **P<0.05** |
| **CD5** | 7.74, 0.68, 0.06 | 8.13, 0.92, 0.12 | **P<0.05** |
| **CD8A** | 11.95, 0.97, 0.13 | 12.34, 1.14, 0.13 | **P<0.05** |
| **CST5** | 6.67, 1.19, 0.11 | 7.15, 1.19, 0.17 | **P<0.05** |
| **FGF-5** | 3.06, 0.30, 0.08 | 3.28, 0.74, 0.09 | **P<0.05** |
| **Flt3L** | 10.17, 0.63, 0.08 | 9.66, 0.81, 0.11 | **P<0.05** |
| **IL-10RB** | 8.77, 0.34, 0.03 | 8.92, 0.41, 0.05 | **P<0.05** |
| **PD-L1** | 7.43, 0.64, 0.06 | 7.83, 0.63, 0.11 | **P<0.05** |
| **SCF** | 8.02, 0.66, 0.06 | 8.18, 0.73, 0.10 | **P<0.05** |
| **VEGFA** | 12.08, 0.52, 0.07 | 12.47, 0.74, 0.12 | **P<0.05** |
| ADA | 7.09, 0.80, 0.10 | 7.17, 0.92, 0.14 | ns |
| AXIN1 | 4.95, 1.49, 014 | 5.18, 1.50, 0.17 | ns |
| CASP-8 | 3.67, 0.90, 0.13 | 3.84, 0.75, 0.13 | ns |
| CCL11 | 9.70, 1.06, 0.10 | 9.75, 0.79, 0.10 | ns |
| CCL19 | 11.13, 1.54, 0.14 | 11.24, 2.14, 0.23 | ns |
| CCL20 | 11.22, 1.71, 0.15 | 11.79, 2.79, 0.26 | ns |
| CCL25 | 8.59, 0.61, 0.08 | 8.70, 0.73, 0.09 | ns |
| CCL28 | 4.36, 0.67, 0.08 | 4.39, 0.55, 0.10 | ns |
| CCL3 | 7.85, 1.09, 0.13 | 8.29, 1.17, 0.17 | ns |
| CCL4 | 7.36, 0.71, 0.15 | 7.47, 1.04, 0.19 | ns |
| CD244 | 7.52, 0.90, 0.08 | 7.69, 0.84, 0.09 | ns |
| CD6 | 6.77, 0.98, 0.10 | 6.85, 0.80, 0.11 | ns |
| CDCP1 | 5.21, 0.80, 0.09 | 5.17, 1.75, 0.20 | ns |
| CSF-1 | 11.26, 0.23, 0.02 | 11.28, 0.25, 0.03 | ns |
| CXCL1 | 9.41, 1.13, 0.15 | 9.07, 1.93, 0.27 | ns |
| CXCL10 | 11.09, 1.58, 0.15 | 10.91, 1.14, 0.19 | ns |
| CXCL11 | 9.09, 1.51, 0.14 | 8.72, 1.89, 0.24 | ns |
| CXCL5 | 7.85, 2.20, 0.22 | 7.26, 1.75, 0.22 | ns |
| CXCL6 | 10.49, 1.29, 0.13 | 10.72, 1.91, 0.22 | ns |
| CXCL9 | 8.68, 2.05, 0.16 | 8.95, 1.54, 0.20 | ns |
| DNER | 9.17, 0.38, 0.06 | 9.12, 0.45, 0.06 | ns |
| EN-RAGE | 4.09, 2.05, 0.20 | 4.33, 1.53, 0.19 | ns |
| FGF-19 | 10.22, 1.57, 0.19 | 10.24, 2.75, 0.30 | ns |
| FGF-21 | 8.62, 3.98, 0.32 | 9.40, 4.49, 0.43 | ns |
| GDNF | 3.81, 0.91, 0.09 | 3.81, 0.75, 0.12 | ns |
| HGF | 11.82, 1.30, 0.13 | 12.00, 1.45, 0.14 | ns |
| IFN-ɣ | 7.74, 2.10, 0.19 | 7.56, 1.81, 0.28 | ns |
| IL10 | 5.32, 0.96, 0.19 | 5.31, 1.40, 0.16 | ns |
| IL-10RA | 2.67, 0.52, 0.06 | 2.86, 0.47, 0.09 | ns |
| IL-12B | 8.20, 1.50, 0.14 | 8.27, 1.74, 0.23 | ns |
| IL-17A | 5.11, 0.92, 0.12 | 4.86, 1.08, 0.21 | ns |
| IL18 | 10.48, 0.98, 0.14 | 10.77, 1.35, 0.22 | ns |
| IL-18R1 | 9.67, 0.70, 0.07 | 9.80, 1.01, 0.13 | ns |
| IL6 | 7.55, 1.37, 0.17 | 6.89, 2.57, 0.29 | ns |
| IL7 | 2.68, 0.58, 0.08 | 2.71, 0.56, 0.08 | ns |
| IL8 | 8.14, 1.69, 0.18 | 8.28, 2.05, 0.27 | ns |
| LAP TGF-β-1 | 9.02, 0.53, 0.06 | 9.17, 0.79, 0.08 | ns |
| LIF-R | 5.38, 0.57, 0.06 | 5.54, 0.52, 0.07 | ns |
| MCP-1 | 12.95, 0.85, 0.09 | 13.06, 1.39, 0.16 | ns |
| MCP-2 | 9.87, 1.08, 0.10 | 9.74, 0.96, 0.13 | ns |
| MCP-3 | 2.89, 0.55, 0.08 | 3.16, 1.15, 0.17 | ns |
| MCP-4 | 1.372, 1.20, 0.13 | 1.356, 1.50, 0.21 | ns |
| MMP-1 | 14.55, 1.22, 0.13 | 14.60, 1.33, 0.17 | ns |
| MMP-10 | 10.63, 1.25, 0.11 | 10.72, 0.96, 0.13 | ns |
| NT-3 | 3.45, 0.43, 0.06 | 3.56, 0.74, 0.11 | ns |
| OPG | 11.54, 0.64, 0.06 | 11.67, 0.69, 0.08 | ns |
| OSM | 5.05, 1.09, 0.14 | 5.44, 1.76, 0.25 | ns |
| SIRT2 | 4.60, 0.99, 0.14 | 4.80, 1.32, 0.18 | ns |
| SLAMF1 | 5.13, 0.59, 0.08 | 5.22, 1.09, 0.11 | ns |
| ST1A1 | 3.12, 1.02, 0.12 | 3.40, 0.88, 0.11 | ns |
| STAMBP | 5.53, 0.83, 0.11 | 5.71, 0.79, 0.11 | ns |
| TNF | 5.51, 0.75, 0.08 | 5.71, 0.97, 0.12 | ns |
| TNFB | 6.12, 0.83, 0.09 | 6.05, 1.04, 0.13 | ns |
| TNFRSF9 | 7.81, 0.76, 0.11 | 8.14, 1.19, 0.18 | ns |
| TNFSF14 | 6.69, 0.78, 0.08 | 6.43, 1.15, .014 | ns |
| TRAIL | 7.61, 0.54, 0.05 | 7.52, 0.57, 0.09 | ns |
| TRANCE | 4.52, 1.17, 0.12 | 4.77, 0.76, 0.13 | ns |
| TWEAK | 9.03, 1.16, 0.10 | 9.13, 0.66, 0.09 | ns |
| uPA | 11.88, 0.74, 0.07 | 12.00, 0.95, 0.10 | ns |

**SI Table 4. Number of proteins detected by Olink in patients with AD and ACLF with high or low FT3.**

| **Target** | **High FT3** | **Low FT3** | **p value** |
| --- | --- | --- | --- |
|  | (median, IQR, SEM  [NPX value]) | (median, IQR, SEM  [NPX value])2 |  |
| **CCL23** | 11.99, 0.85, 0.10 | 12.34, 0.65, 0.08 | **P<0.01** |
| **VEGFA** | 12.03, 0.79, 0.10 | 12.32, 0.59, 0.08 | **P<0.01** |
| **CD40** | 13.04, 0.77, 0.12 | 13.43, 07.5, 0.10 | **P<0.05** |
| **CXCL9** | 8.24, 1.66, 0.18 | 9.06, 1.57, 0.16 | **P<0.05** |
| **FGF-23** | 5.28, 2.83, 0.32 | 6.20, 2.89, 0.27 | **P<0.05** |
| **IL-17C** | 4.71, 1.66, 0.18 | 5.66, 1.50, 0.16 | **P<0.05** |
| **TGF-α** | 4.59, 0.93, 0.10 | 4.79, 0.79, 0.11 | **P<0.05** |
| **TNF** | 5.51, 0.66, 0.08 | 5.74, 0.93, 0.09 | **P<0.05** |
| **TNFB** | 6.25, 0.76, 0.10 | 5.94, 0.95, 0.10 | **P<0.05** |
| **TRANCE** | 4.81, 0.97, 0.12 | 4.41, 1.10, 0.12 | **P<0.05** |
| **TWEAK** | 9.28, 0.81, 0.11 | 8.93, 0.77, 0.08 | **P<0.05** |
| 4E-BP1 | 9.48, 1.76, 0.17 | 10.00, 1.09, 0.12 | ns |
| ADA | 7.31, 0.72, 0.12 | 7.14, 0.80, 0.10 | ns |
| AXIN1 | 4.97, 1.49, 0.16 | 5.02, 1.44, 0.14 | ns |
| CASP-8 | 3.80, 0.85, 0.13 | 3.79, 0.80, 0.13 | ns |
| CCL11 | 9.65, 0.93, 0.12 | 9.17, 0.82, 0.08 | ns |
| CCL19 | 11.08, 1.59, 0.17 | 11.20, 1.97, 0.17 | ns |
| CCL20 | 11.53, 1.68, 0.18 | 11.24, 2.38,0.20 | ns |
| CCL25 | 8.59, 0.54, 0.09 | 8.69, 0.69, 0.08 | ns |
| CCL28 | 4.38, 0.63, 0.09 | 4.32, 0.57, 0.08 | ns |
| CCL3 | 7.85, 1.18, 0.16 | 8.27, 1.01, 0.12 | ns |
| CCL4 | 7.48, 0.61, 0.17 | 7.43, 1.04, 0.16 | ns |
| CD244 | 7.70, 0.94, 0.10 | 7.257, 0.74, 0.08 | ns |
| CD5 | 7.74, 0.59, 0.10 | 8.09, 0.82, 0.08 | ns |
| CD6 | 6.84, 0.84, 0.11 | 6.66, 0.89, 0.10 | ns |
| CD8A | 12.17, 0.91, 0.14 | 12.11, 1.22, 0.13 | ns |
| CDCP1 | 5.23, 1.05, 0.13 | 5.27, 0.95, 0.12 | ns |
| CSF-1 | 11.27, 0.19, 0.2 | 11.26, 0.26, 0.03 | ns |
| CST5 | 6.74, 1.27, 0.13 | 6.85, 1.22, 0.14 | ns |
| CX3CL1 | 6.30, 0.84, 0.11 | 6.35, 0.69, 0.09 | ns |
| CXCL1 | 9.37, 1.21, 0.19 | 9.38, 1.64, 0.19 | ns |
| CXCL10 | 10.90, 1.26, 0.17 | 11.09, 1.17, 0.16 | ns |
| CXCL11 | 8.81, 0.97, 0.14 | 9.08, 1.88, 0.19 | ns |
| CXCL5 | 7.55, 1.88, 0.22 | 7.54, 2.12, 0.24 | ns |
| CXCL6 | 10.73, 1.07, 0.14 | 10.71, 1.62, 0.17 | ns |
| DNER | 9.19, 0.44, 0.07 | 9.09, 1.57, 0.16 | ns |
| EN-RAGE | 4.12, 2.02, 0.24 | 4.30, 1.59, 0.16 | ns |
| FGF-19 | 10.29, 2.19, 0.24 | 10.18, 1.77, 0.21 | ns |
| FGF-21 | 9.33, 3.20, 0.36 | 9.24, 4.25, 0.36 | ns |
| FGF-5 | 3.01, 0.38, 0.12 | 3.19, 0.30, 0.06 | ns |
| Flt3L | 10.09, 0.72, 0.10 | 9.95, 0.84, 0.08 | ns |
| GDNF | 3.83, 0.67, 0.08 | 3.81, 1.02, 0.11 | ns |
| HGF | 12.06, 1.17, 0.14 | 11.76, 1.39, 0.13 | ns |
| IFN-ɣ | 7.56, 1.64, 0.21 | 7.75, 2.38, 0.24 | ns |
| IL10 | 5.51, 0.98, 0.25 | 5.33, 0.99, 0.13 | ns |
| IL-10RA | 2.70, 0.56, 0.06 | 2.76, 0.53, 0.06 | ns |
| IL-10RB | 8.80, 0.40, 0.04 | 8.87, 0.32, 0.04 | ns |
| IL-12B | 8.23, 1.17, 0.16 | 8.20, 1.76, 0.18 | ns |
| IL-15RA | 3.76, 0.93,0.08 | 4.05, 0.80, 0.08 | ns |
| IL-17A | 5.05, 0.91, 0.13 | 5.11, 1.08, 0.16 | ns |
| IL18 | 10.48, 1.26, 0.19 | 10.75, 0.97, 0.15 | ns |
| IL-18R1 | 9.80, 0.67, 0.11 | 9.64, 0.81, 0.09 | ns |
| IL6 | 7.41, 1.83, 0.24 | 7.63, 1.75, 0.20 | ns |
| IL7 | 2.72, 0.61, 0.09 | 2.66, 0.49, 0.07 | ns |
| IL8 | 8.12, 1.93, 0.22 | 8.37, 1.74, 0.19 | ns |
| LAP TGF-β-1 | 9.09, 0.56, 0.06 | 9.05, 0.72, 0.07 | ns |
| LIF-R | 5.59, 0.55, 0.06 | 5.37, 0.55, 0.06 | ns |
| MCP-1 | 12.90, 1.04, 0.12 | 13.09, 0.92, 0.11 | ns |
| MCP-2 | 9.76, 0.95, 0.12 | 9.72, 1.13, 0.11 | ns |
| MCP-3 | 2.96, 0.54. 0.11 | 3.04, 1.07, 0.11 | ns |
| MCP-4 | 13.81, 1.17, 0.14 | 13.64, 1.37, 0.17 | ns |
| MMP-1 | 14.45, 1.63, 0.17 | 14.75, 0.97, 0.12 | ns |
| MMP-10 | 10.76, 1.37, 0.13 | 10.53, 1.07, 0.11 | ns |
| NT-3 | 3.58, 0.33, 0.06 | 3.39, 0.57, 0.08 | ns |
| OPG | 11.63, 0.55, 0.07 | 11.67, 0.70, 0.07 | ns |
| OSM | 5.26, 1.16, 0.15 | 5.26, 1.27, 0.20 | ns |
| PD-L1 | 7.50, 0.72, 0.09 | 7.71, 0.58, 0.08 | ns |
| SCF | 8.00, 0.76, 0.08 | 8.15, 0.5, 0.07 | ns |
| SIRT2 | 4.62, 1.24, 0.15 | 4.76, 1.07, 0.15 | ns |
| SLAMF1 | 5.20, 0.63, 0.09 | 5.11, 1.01, 0.09 | ns |
| ST1A1 | 3.12, 1.04, 0.13 | 3.35, 0.83, 0.11 | ns |
| STAMBP | 5.53, 0.88, 0.11 | 5.61, 0.77, 0.11 | ns |
| TNFRSF9 | 7.89, 0.86, 0.16 | 8.03, 0.87, 0.12 | ns |
| TNFSF14 | 6.50, 0.82, 0.11 | 6.70, 0.84, 0.09 | ns |
| TRAIL | 7.62, 0.50, 0.05 | 7.57, 0.57, 0.06 | ns |
| uPA | 12.06, 0.72, 0.08 | 11.89, 0.62, 0.08 | ns |

**SI Figure 1. Association between thyroid hormones and stages of liver cirrhosis: impact of etiology.**

Violin blots of thyroid hormone concentrations are shown for patients with alcoholic liver cirrhosis compared to patients with other etiologies of liver cirrhosis.

Graphs show value distribution with median (black dashed line) ± quartiles (colored dashed lines). -way ANOVA or Kruskal-Wallis test were used as appropriate after Normality Test. *P ≤ 0.05, **P ≤ 0.01, ***P ≤ 0.001, ****P≤ 0.0001

ACLF, acute-on-chronic liver failure. AD, acute decompensation; Comp., compensated cirrhosis; FT_3_, free triiodothyronine; FT_4_, free tetraiodothyronine; TSH, thyroid-stimulating hormone.
